# Supplementary material for: Identification of Familial Hodgkin Lymphoma Predisposing Genes Using Whole Genome Sequencing
Source: Front Bioeng Biotechnol. 2020 Mar 6;8:179. doi: 10.3389/fbioe.2020.00179 (PMC7067901; doi:10.3389/fbioe.2020.00179)
Supplement: Supplementary file 1 [file Data_Sheet_1.PDF]

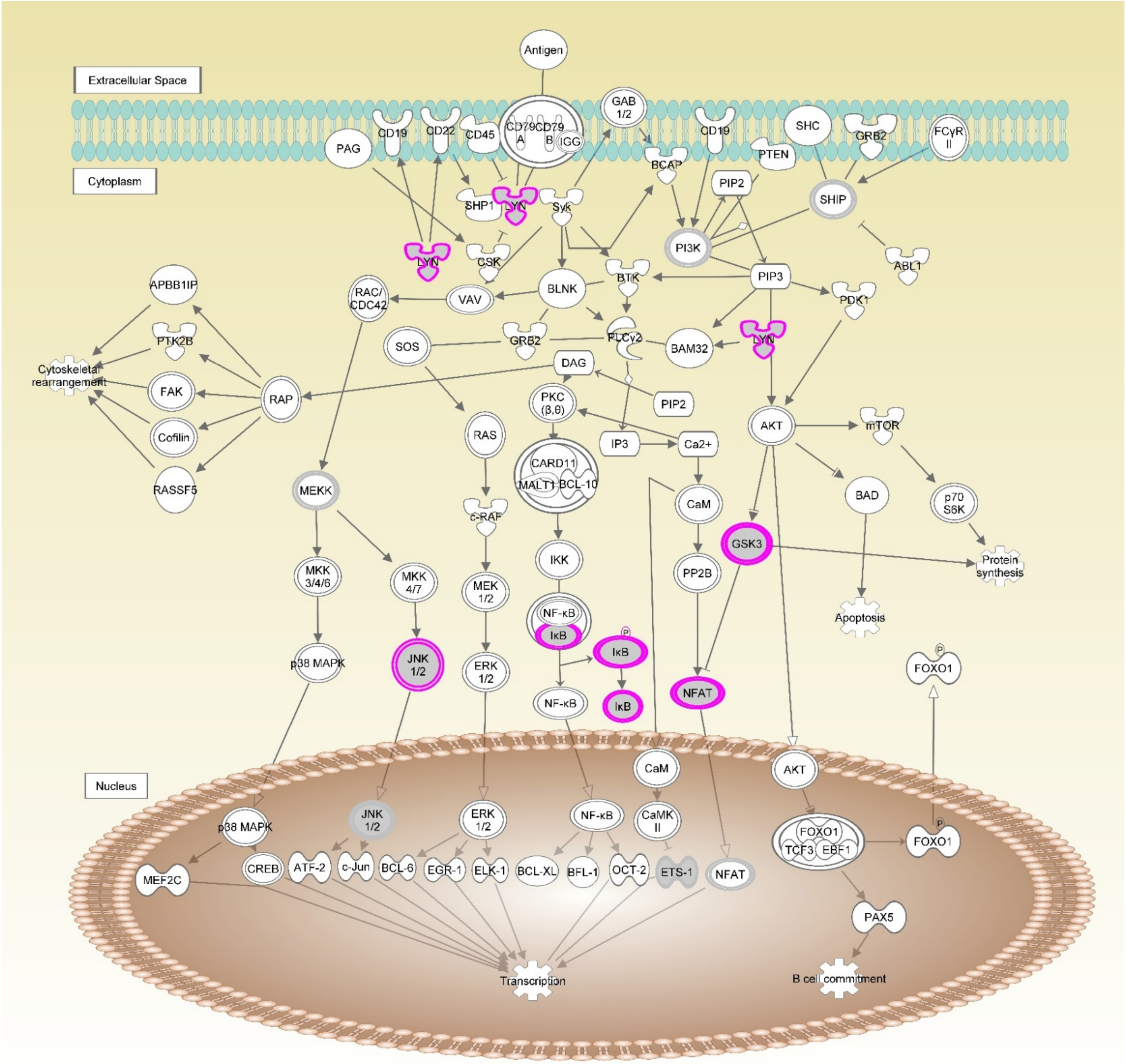

© 2000-2019 QIAGEN. All rights reserved.

Supplementary Figure 1A. B-Cell Receptor Signaling

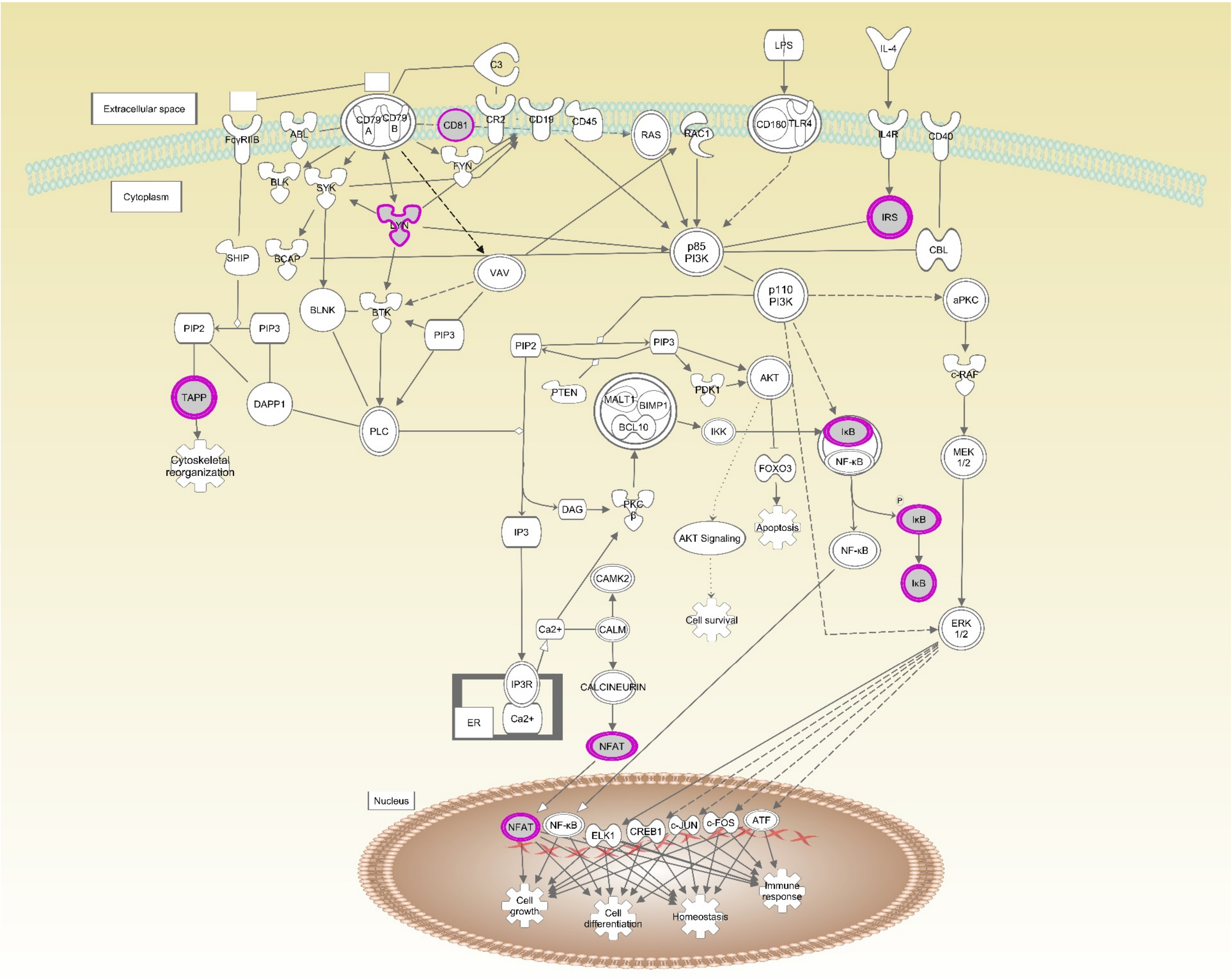

© 2000-2019 QIAGEN. All rights reserved.

Supplementary Figure 1B. PI3K Signaling in B Lymphocytes
